# Effect of aridity and dune type on rhizosphere soil bacterial communities of *Caragana microphylla* in desert regions of northern China

**DOI:** 10.1371/journal.pone.0224195

**Published:** 2019-10-18

**Authors:** Jiangli Gao, Yang Luo, Yali Wei, Yaolong Huang, Hua Zhang, Wenliang He, Hongmei Sheng, Lizhe An

**Affiliations:** 1 Ministry of Education Key Laboratory of Cell Activities and Stress Adaptations, School of Life Sciences, Lanzhou University, Lanzhou, China; 2 The College of Forestry, Beijing Forestry University, Beijing, China; Universite Paris-Sud, FRANCE

## Abstract

Understanding the response of soil properties and bacterial communities in rhizosphere soil to aridity and dune types is fundamental to desertification control. This study investigated soil properties and bacterial communities of both rhizosphere and bulk soils of *Caragana microphylla* from four sites with different aridity indices, and one site with three different types of dunes. All sites were located in the desert regions of northern China. The results indicated that compared with the bulk soil, the soil nutrient content of rhizosphere, especially the content of total phosphorus, was generally significantly improved in different desertification environments. The bacterial richness and diversity were also higher than those of bulk soil, especially in arid regions and fixed dunes. Firmicutes, Actinobacteria, Proteobacteria, and Acidobacteria were the most dominant phyla in all samples. The regression analyses showed that at different sites, soil total organic C, total N, Na^+^, and total P played key roles in determining the bacterial community structure while total organic carbon, electronic conductivity, pH and total phosphorus were the dominant factors at the different dunes. The results further revealed that the dominant phyla strongly affected by environmental factors at different sites were Acidobacteria, Gemmatimonadetes, and Actinobacteria among which, Acidobacteria and Gemmatimonadetes were negatively correlated with Na^+^ content. At different types of dunes, Actinobacteria, Planctomycetes, and Gemmatimonadetes were particularly affected by environmental factors. The increased abundance of Actinobacteria in the rhizosphere soil was mainly caused by the decreased soil pH.

## Introduction

Desertification is a land degradation process caused by climate change and human activities in arid, semi-arid, and partially semi-humid areas [1], which directly threatens the economic, social, and environmental security of both regions and countries [2,3]. China is one of the countries most severely affected by desertification worldwide [4]. In arid and semi-arid areas of China, the degradation of cultivated land and grassland, the reactivation of fixed dunes and semi-fixed dunes are the main forms of land degradation, and the increase of sediment transport caused by the decrease of vegetation is the main reason for the above situation [5,6]. Therefore, in recent decades, China has made considerable efforts to combat desertification with remarkable results such as vegetation restoration in desertification affected areas [7]. Some plants and microorganisms present strong stress resistance, and are considered critical to the process of vegetation restoration and soil improvement.

*Caragana microphylla*, a species of the genus *Caragana* in the Leguminosae family, is an important shrub for restoration in desertification areas. *C*. *microphylla* is widely distributed in arid, semi-arid, and semi-humid regions of north China [8], and is highly tolerant to drought, hypersalinity, and extreme cold in poor or sandy soils. It has been widely used as a sand barrier for vegetation restoration of desertified land [9].

Soil microorganisms have contributed considerably ton regulating soil fertility, plant health, and nutrient cycling such as carbon and nitrogen [10], especially rhizomicrobiome, which can improve the adaptability of plants to extreme environments [11]. Studies have shown that the rhizosphere of plants could effectively enrich the bacterial communities with multiple plant growth promoting (PGP) activities and stress resistance capabilities under drought conditions [12]. The diversity and functional redundancy of these related plant growth promoting rhizobacteria (PGPR) make them actively participate in the adaptation of plants to different desertification environments [13]. There are complex relationships among these microbial communities, and their composition and abundance depend on many factors, such as soil properties, plant species, and the surrounding abiotic environment. Abiotic environmental factors such as drought and dune type have been shown to have a dramatic impact on bacterial communities, but little is known about the underlying causes behind the observed changes in microbial abundance [14,15,16]. Recently, increasing attention has been drawn to the interaction between plants and their rhizosphere bacterial communities. Some studies have shown that soil and plants strongly influence the composition of rhizosphere microbiome [17,18]. Specific soil properties such as pH, concentrations of N, P, K, and other mineral nutrients show different effects on the composition of rhizosphere bacterial community [17,19–21]. The effects of different tree ages of *C*. *microphylla* and other species of *Caragana* on rhizosphere bacteria community were also explained [22,23]. Different aridity and dune types also have significant effects on rhizosphere bacterial communities [24,16]. Researches in the Loess Plateau and the Namib Desert has led to an in-depth understanding of the relationship between microbial nutrient limits and microbial community structure in arid and semi-arid ecosystems, and the response of microbial communities to nutrient limits [25,26]. The 16S RNA gene diversity of soil bacteria was studied by high-throughput sequencing of the grassland sample belt in northern China, and the results showed that the aridity index was the most significant factor affecting the composition and diversity of the bacterial community [27]. Studies have also shown that the composition of the microbial communities associated with Cacti growing in arid and semi-arid areas was mainly influenced by plant compartment, while plant species, sites and seasons played a secondary role [28]. In degraded sandy grasslands in semi-arid regions (even degraded to moving dunes), the community structure of soil bacteria can be reversibly restored by planting shrubs or semi-shrubs (including *C*. *microphylla*) [29]. However, in-depth investigations of rhizosphere bacterial communities of *C*. *microphylla* on large geographical scale and different types of sand dunes have yet to be conducted. Through the investigation of the soil bacterial community formed in the rhizosphere soil of *C*. *microphylla*, the interaction between soil and plant can be better understood, the mechanism of vegetation protection and restoration under the desertification environment with different aridity indexes and different dune types can be understood, and the restoration of the degraded ecosystem can be evaluated.

It is imperative to obtain a better understanding of these interactions because rhizosphere bacteria could improve stress resistance and promote the growth of *C*. *microphylla* in different desertification environments [30]. Furthermore, the effect of aridity index (AI) and sand dune types on soil bacterial communities have not been comprehensively studied. In this study, we chose five *C*. *microphylla* habitats to investigate the above phenomena in desert regions of northern China. Four of these sites with increasing AI were used to study the effects of different AIs and one site was used to study the effects of different dune types. Furthermore, we performed deep sequencing using Illumina 16S rRNA amplicons to reveal the composition of the rhizosphere bacterial community. The objectives were to (1) assess soil property changes under the influence of rhizosphere, aridity, and dune type, (2) determine the composition and diversity of the bacterial community across *C*. *microphylla* habitats, (3) explore possible soil properties leading to changes in soil bacterial community.

To our knowledge, this study is the first to study the effect of aridity index and dune type on the diversity of the rhizosphere soil bacterial community of *C*. *microphylla* in different desertification environments in northern China by using high-throughput sequencing. It was also the first attempt to reveal the important environmental factors affecting the rhizosphere soil bacterial community of *C*. *microphylla* in the study area. This study could provide theoretical basis for vegetation restoration and stability of desert ecosystem in desertification areas.

## Materials and methods

### Study sites and experimental design

**Fig 1:**
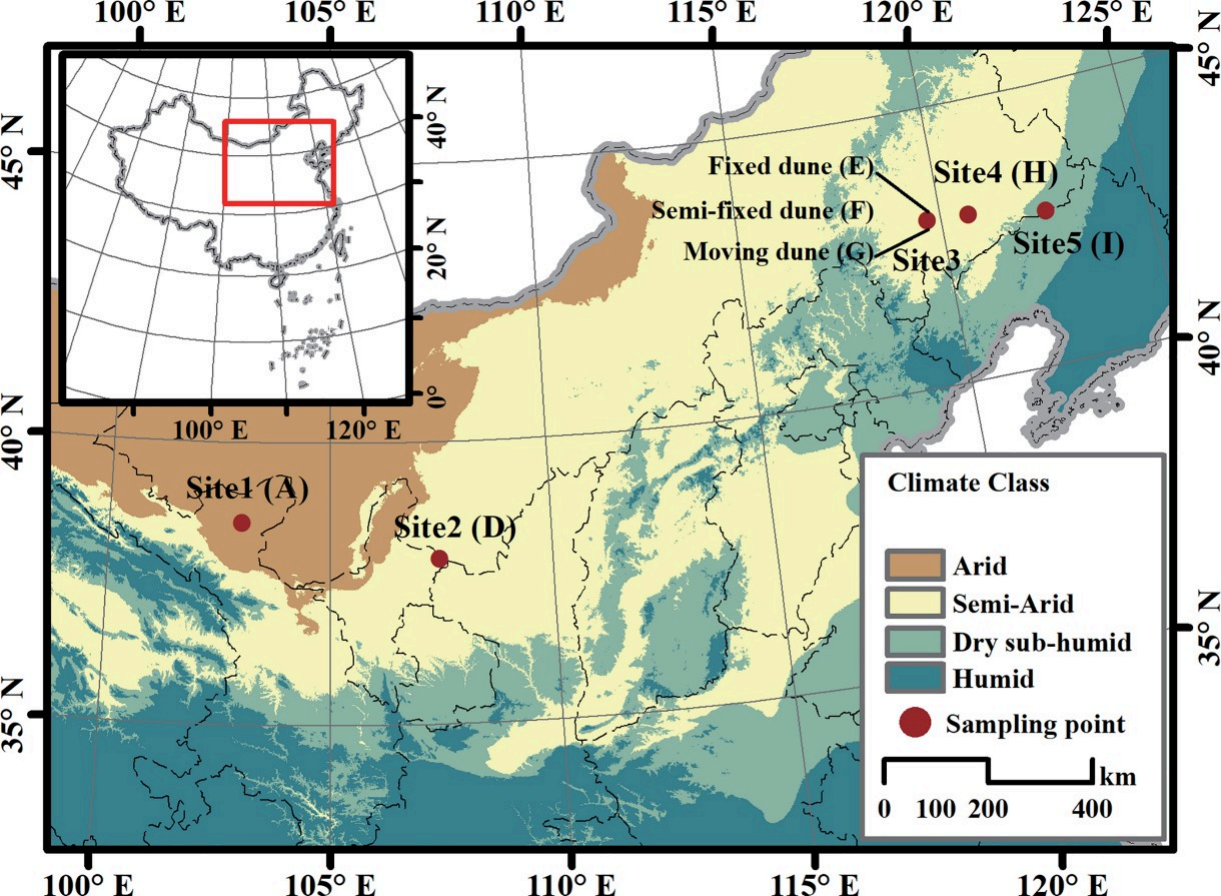
Location of the study sites.

The bulk and rhizosphere soil samples from *C*. *microphylla* habitats were collected at the five sites in mid-May 2014. Permissions for collection of samples were from the Comprehensive Experimental Station of Minqin Desertification Control, Chinese Academy of Forestry (site 1); the Cold and Arid Regions Environmental and Engineering Research Institute, Chinese Academy of Sciences. (site 2, 3, 4); and the Liaoning Institute of Sand Fixation and Afforestation (site 5). These five sites spanned the desertification region of northern China (Fig 1). The AI data in Fig 1 was the annual average over the 1950–2000 period, which used data from the WorldClim Global Climate Data [31]. AI = MAP/MAE, where MAP is mean annual precipitation and MAE is mean annual potential evapo-transpiration.

Fig 1 shows that the AI values at sites A, D, H and I were 0.1, 0.26, 0.42, and 0.55, respectively. According to the generalized climate classification scheme for global-aridity values [32], site A was an arid area (AI values of 0.03 to 0.2), sites D and H were semi-arid desertification areas (AI values of 0.2 to 0.5), and site I was a dry sub-humid desertification area (AI values of 0.5 to 0.65). The dune types of these four sites were all fixed. Furthermore, another site located near site H was a semi-arid desertification area (AI value is 0.38). This site included three different type of dunes, *i*.*e*., fixed (E), semi-fixed (F), and moving (G) dunes.

Two relatively separate but closely related trials were designed and conducted to obtain physicochemical data and bacteria community characteristics of rhizosphere and bulk soil. Trial 1 was designed to examine the differences in the above parameters across the four sites with different AI values including sites D, H and I. Trial 2 was focused on evaluating the differences in the above parameters among three dune types (fixed, semi-fixed, and moving dunes).

### Soil material and sampling

*C*. *microphylla* rhizosphere soil was extracted by digging 50 cm deep along the roots and separating the loosely adhering soil from the rhizosphere soil [33]. Each replicate rhizosphere soil sample consisted of three randomly selected adhering soil samples taken from three plants (approximately 300 g from each individual). Each bulk soil sample was collected from an area approximately 200 cm from the plants. Three replicate samples were taken from the rhizosphere and bulk soils and were all placed in aseptic aluminum cans and immediately transported in a cooling box to the laboratory and stored at −20°C.

### Soil physicochemical analyses

All samples were analyzed for pH, electronic conductivity (EC), total organic carbon (TOC), total nitrogen (TN), total phosphorus (TP), Ca^2+^, K^+^, and Na^+^.pH and EC were determined at a soil to water ratio of 1:5. TOC was measured using the potassium dichromate oxidation method. TN content was analyzed using dry combustion using a C/N analyzer (GmbH VarioEL, Elementar Analysen System, Langenselbold, Germany). The total P was measured using the phosphoric acid molybdenum antimony colorimetric method. Ca^2+^, K^+^, and Na^+^ contents were determined using flame spectrophotometry. All measurements were performed according to the method of Smith, 1983 [34].

### DNA extraction and polymerase chain reaction (PCR) amplification

The total DNA of bacteria from the soil samples was extracted using the E.Z.N.A.^®^ soil DNA kit (Omega Bio-tek, Norcross, GA, US). The final DNA concentration and purification were determined using NanoDrop 2000 UV-vis spectrophotometer (Thermo Scientific, Wilmington, DE, USA), and DNA quality was checked using 1% agarose gel electrophoresis. The V4-V5 hypervariable regions of the bacterial 16S rRNA gene were amplified using primers 515F (5ʹ-GTGCCAGCMGCCGCGG-3ʹ) and 907R (5ʹ-CCGTCAATTCMTTTRAGTTT-3ʹ). The V4-V5 regions of each sample were amplified using polymerase chain reaction (PCR, GeneAmp 9700, ABI, USA). The resulting PCR products were extracted from a 2% agarose gel and further purified using the AxyPrep DNA gel extraction kit (Axygen Biosciences, Union City, CA, USA) and quantified using QuantiFluor ^™^ -ST (Promega, USA) according to the manufacturer’s protocol.

### Illumina MiSeq sequencing

Purified amplicons were pooled in equimolar and paired-end sequenced (2 × 300 bp) using an Illumina MiSeq platform (Illumina, San Diego, CA, USA) according to the standard protocols of the Majorbio Bio-Pharm Technology Co. Ltd. (Shanghai, China). The raw reads were deposited into the NCBI Sequence Read Archive (SRA) database (Accession Number: SRR8772877–SRR8772882).

### MiSeq sequencing data processing and analysis

Raw fastq files were quality-filtered by Trimmomatic and merged by FLASH as reported [35] with the following criteria: (i) The reads were truncated at any site receiving an average quality score <20 over a 50 bp sliding window. (ii) Sequences whose overlap being longer than 10 bp were merged according to their overlap with mismatch no more than 2 bp. (iii) Sequences of each sample were separated according to barcodes (exactly matching) and Primers (allowing 2 nucleotide mismatching), and reads containing ambiguous bases were removed.

Operational taxonomic units (OTUs) were clustered with a 97% similarity cutoff using UPARSE (version 7.1 http://drive5.com/uparse/) with a novel “greedy” algorithm that performs chimera filtering and OTU clustering simultaneously. The taxonomy of each 16S rRNA gene sequence was analyzed using the RDP classifier algorithm (http://rdp.cme.msu.edu/) against the Silva (SSU123) 16S rRNA database using confidence threshold of 70%.

Rarefaction curves and alpha diversity indices including richness (Chao and Ace), microbial community diversity (Shannon index), and coverage were calculated using Mothur [36]. Beta diversity indices between samples using the weighted UniFrac distances were calculated using the QIIME. PERMANOVA (Permutational Multivariate Analysis Of Variance) was used to detect the differences between community groups based on Bray-Curtis distances. To identify the differences in bacterial community composition, the weighted UniFrac distance was calculated to present non-metric multi-dimensional scaling (NMDS) based on Bray-Curtis distances using the metaMDS function of the vegan R package [37]. The process forms two-dimensional maps (NMDS1 and NMDS2) based on the difference of samples. To further explore the relationship between bacterial communities and soil properties, Redundancy analysis (RDA) and mental test were performed to study the relationship between the most abundant bacteria and soil properties. One-way ANOVA was conducted to analyze the significance of the difference with Duncan, differences were considered significant at P < 0.05, and Spearman's rank-order correlation was performed using the statistical package for the social sciences (SPSS) version 20.0 (SPSS Inc., Chicago, USA). Spearman correlation heatmap was drawn based on the Spearman's rank-order correlation using the R software.

## Results

### Abiotic soil properties

Soil properties of the rhizosphere and bulk soil from four sites and three types of dunes are presented in Tables 1 and 2. The Kolmogorov-Smirnov normality test indicated that the data were normally distributed (P value of pH = 0.28, P value of EC = 0.22, P value of TOC = 0.12, P value of TN = 0.60, P value of TP = 0.08, P value of Ca^2+^ = 0.06, P value of K^+^ = 0.09, P value of Na^+^ = 0.08). Generally, with increasing AI, the rhizosphere soil nutrient content showed an ascending trend, and the soil pH, Ca^2 +^, and Na^+^ content declined (Table 1). Furthermore, the soil properties of rhizosphere soil were generally different from bulk soil, in particular, the content of TP in rhizosphere soil was significantly higher than bulk soil (Table 1). The contents of TOC, TN, and TP in the rhizosphere soil were significantly higher than those in the bulk were soil at three types of dunes, whereas the pH and ion contents such as Ca^2+^, K^+^, and Na^+^ were significantly lower than those of the bulk soil were. In particular, the TOC and TP in the rhizosphere soil of the fixed dune were significantly higher than those of semi-fixed and moving dunes were (Table 2).

**Table 1:** Soil chemical properties of four sites.

Samples	pH	EC (μs/cm)	TOC (g/kg)	TN (mg/kg)	TP (mg/kg)	Ca^2+^ (mg/kg)	K^+^ (mg/kg)	Na^+^ (mg/kg)
AC	8.84b	59.93a	1.543d	209.22de	12.60e	2154.08b	118.17d	103.21c
AM	8.74b	54.83b	1.133e	212.26de	24.39d	4493.59a	114.43d	315.18a
DC	9.46a	37.50d	0.893e	135.30f	13.12e	2102.07b	143.04c	135.25b
DM	8.63b	31.30e	1.453d	181.50e	23.61d	2335.37b	150.31bc	48.43d
HC	7.10c	41.53c	4.10b	306.50b	26.23d	631.13c	154.22b	54.78d
HM	7.08c	41.93c	4.033b	408.07a	37.92c	674.38c	197.47a	53.72d
IC	6.75d	52.90b	7.173a	280.53bc	44.43b	347.00cd	109.90d	51.06d
IM	6.71d	40.93c	3.483c	251.60cd	52.69a	234.95d	101.04e	37.01e
*F*	427.70	129.07	132.04	17.88	107.12	185.58	126.79	1561.20
*P*	***	***	***	***	***	***	***	***

Values are means of three replicates.

Means in the column followed by different letters are significantly different (P < 0.05).

*** P < 0.001.

A: Site A; D: Site D; H: Site H; I: Site I; C: Bulk soil; M: Rhizosphere soil.

**Table 2:** Soil chemical properties of three types of dunes.

Samples	pH	EC (μs/cm)	TOC (g/kg)	TN (mg/kg)	TP (mg/kg)	Ca^2+^ (mg/kg)	K^+^ (mg/kg)	Na^+^ (mg/kg)
EC	7.48ab	9.11c	0.94c	75.97c	4.72bc	384.03a	207.56a	112.47a
EM	6.75cd	19.18a	2.03a	219.27a	5.63a	180.38c	84.10e	44.50e
FC	7.04bc	15.16b	0.51d	233.73a	4.99b	378.82a	101.37d	48.23de
FM	6.17d	10.01c	1.03c	231.33a	4.48bc	205.21c	111.00c	62.59c
GC	7.89a	9.23c	0.78cd	162.23b	3.30d	383.63a	134.12b	71.29b
GM	7.13bc	9.95c	1.47b	222.77a	4.37c	232.18b	84.03e	51.84d
*F*	15.71	62.04	12.08	12.01	16.97	313.18	587.26	364.64
*P*	***	***	***	***	***	***	***	***

Values are means of three replicates.

Means in the column followed by different letters are significantly different (P < 0.05).

***P < 0.001.

E: Fixed dune; F: Semi-fixed dune; G: Moving dune; C: Bulk soil; M: Rhizosphere soil.

### General characteristics of MiSeq sequencing data

Illumina Miseq sequencing results showed that 1,241,997 sequences and 3,783 OTUs were obtained from 42 soil samples. From each sample, 13,413 to 18,411 high-quality sequences were presented, indicating an average of 16,655 sequences per sample. Effective sequences with a length of 380 to 400 bp accounted for 99.72% of the total sequences. The rarefaction curves based on OTU tended to be saturated with increased sequencing amounts, indicating that the sequencing amount could cover most species in soil samples and could be used for data analysis (S1 Fig).

### Soil bacterial community composition

**Fig 2:**
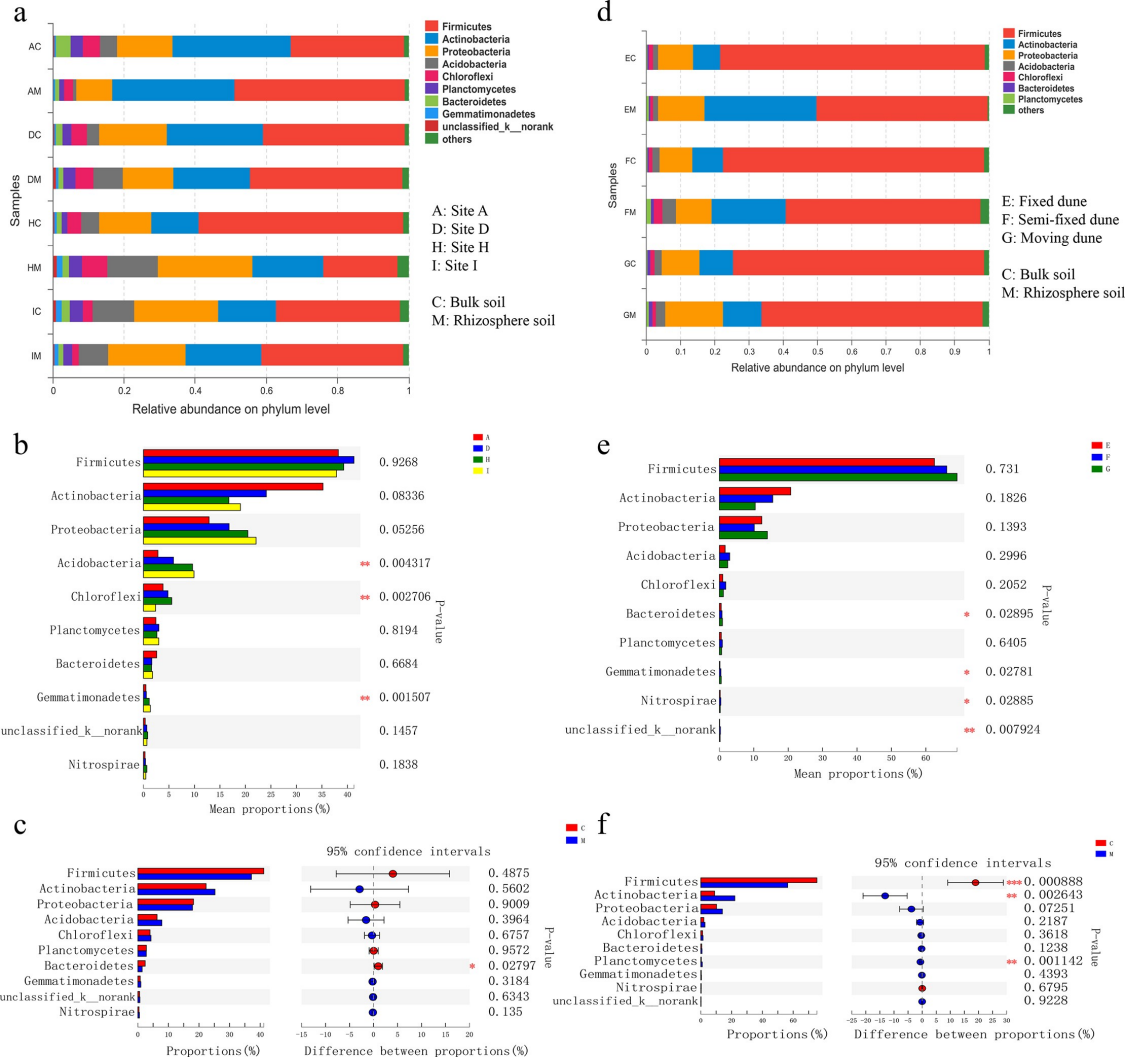
Relative abundance of bacterial phyla. Across (a) four sites and (d) three types of dunes. (b) and (e) are significance test analysis between four sites and three types of dunes based on one-way ANOVA. (c) and (f) are significance test analysis between rhizosphere and bulk soil of four sites and three types of dunes based on Student’s t-test. Data are presented as mean, n = 3. * P < 0.05, ** P < 0.01, *** P < 0.001.

Bacterial OTUs obtained at four sites with different AIs were assigned to 32 phyla, 330 families, and 621 genera. The dominant phyla were Firmicutes, Actinobacteria, Proteobacteria, and Acidobacteria (Fig 2A). Other relatively abundance phyla were Chloroflexi, Planctomycetes, and Bacteroidetes. Gemmatimonadetes were also present in all soil samples. In general, the abundance of Actinobacteria, Acidobacteria, Chloroflexi, and Planctomycetes were presented a higher abundance in the rhizosphere soil. The relative abundance of Acidobacteria, Chloroflexi and Gemmatimonadetes among the four sites were significantly different under the influence of AI, and they showed an upward trend with the increase of AI (Fig 2B). The relative abundance of Bacteroidetes in the rhizosphere soil of the four sites was significantly higher than that in the bulk soil (Fig 2C).

**Fig 3:**
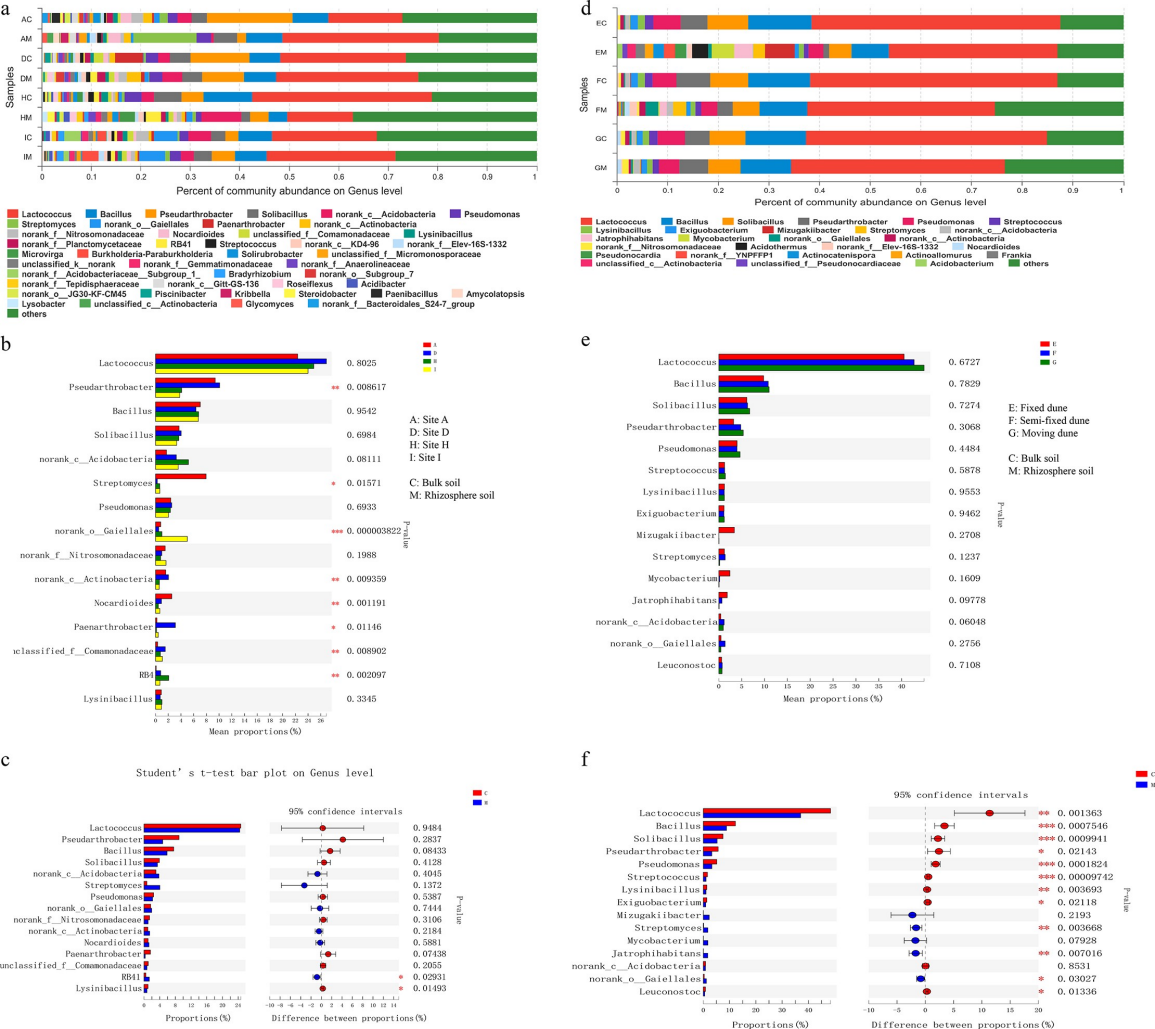
Relative abundance of bacterial genera. Across (a) four sites and (d) three types of dunes. (b) and (e) are significance test analysis between four sites and three types of dunes based on one-way ANOVA. (c) and (f) are significance test analysis between rhizosphere and bulk soil of four sites and three types of dunes based on Student’s t-test. Data are presented as mean, n = 3. * P < 0.05, ** P < 0.01, *** P < 0.001.

At the genera level, *Lactococcus*, *Bacillus*, *Pseudarthrobacter*, *Solibacillus*, *norank_c__Acidobacteria*, *Pseudomonas*, and *Streptomyces* were dominant bacteria in all samples (Fig 3A). There were significant differences in the relative abundance of many dominant genera in four sites with different AI, such as *Pseudarthrobacter*, *Streptomyces*, *norank_o__Gaiellales*, *norank_c__Actinobacteria*, *Nocardioides*, *Paenarthrobacter*, etc (Fig 3B). The relative abundance of RB41 and Lysinibacillus was significantly different between rhizosphere and bulk soil, but they were not the most dominant genera (Fig 3C).

For different dune types, bacterial OTUs were assigned to 34 phyla, 282 families, and 496 genera. The dominant phyla in three dunes were Firmicutes, Actinobacteria, and Proteobacteria (Fig 2D). Acidobacteria, Chloroflexi, Bacteroidetes, and Planctomycetes were also present in all soil samples, but their abundance was lower. The relative abundance of Bacteroidetes, Gemmatimonadetes, Nitrospirae and unclassified_k__norank varies significantly among different types of dunes (Fig 2E). Firmicutes accounted for more than 70% of all bulk soils, and the relative abundance of this phyla was significantly reduced in rhizosphere soils. However, the relative abundance of Actinobacteria and Bacteroidetes in the rhizosphere was significantly higher than that in the bulk soil (Fig 2F).

The genera with the highest relative abundance, *Lactococcus*, *Bacillus*, *Solibacillus*, *Pseudarthrobacter*, *Pseudomonas*, *Streptococcus*, *Lysinibacillus* and *Exiguobacterium* were all significantly higher in the bulk soil than in the rhizosphere soil, and the relative abundance of *Streptomyces* and *Jatrophihabitans* in rhizosphere soil was significantly higher than that in bulk soil (Fig 3D and 3F). However, there was no significant difference in the relative abundance of these most dominant genera between different types of dunes (Fig 3E).

A Venn diagram used to evaluate the distribution of OTUs among the different habitats (S2 Fig) showed numerous common OTUs between rhizosphere and bulk soils. However, at different sites and types of dunes, the proportion of unique OTU in the rhizosphere soil was 11.39% and 17.34%, respectively.

### Soil bacterial diversity across different habitats

Tables 3 and 4 showd the coverage, ACE, Chao1, and Shannon indices in different sites (Table 3, P < 0.05) and different types of dunes (Table 4, P < 0.05), respectively. The coverage of all samples was between 97.36% and 99.62%, indicating that sequencing reads were sufficient for statistical analysis. Chao1 and ACE indexes that represented alpha species richness, and the Shannon index that represented species diversity showed an upward trend from site A to I (Table 3). However, Shannon index were significantly higher in rhizosphere soils than in bulk soils at fixed dune and semi-fixed dune (Table 4).

**Table 3:** Estimated number of observed operational taxonomic units (OTUs), coverage, richness, and diversity at four sites.

Samples	OTU	Coverage (%)	ACE	Chao1	Shannon
AC	824.67c	98.15ab	985.89d	979.36d	4.49ab
AM	866.33c	98.91a	1058.02d	1065.78d	4.00b
DC	1233.00ab	99.00a	1468.96b	1480.76bc	4.36b
DM	1434.33a	98.69a	1653.47ab	1656.08ab	4.60ab
HC	1382.00a	98.55a	1637.42ab	1638.37ab	4.11b
HM	1486.67a	97.36b	1816.98a	1805.56a	5.67a
IC	1428.00a	98.48a	1550.17b	1541.44b	5.21ab
IM	1114.00b	98.53a	1286.01c	1287.73c	4.61ab
*F*	19.32	5.60	47.23	34.87	4.67
*P*	***	**	***	***	**

OTUs, operational taxonomic units; Coverage, Good's nonparametric coverage estimator; ACE, abundance-based coverage estimator; Chao1, richness estimator; Shannon, nonparametric Shannon diversity index.

Values are means of three replicates.

Means in the column followed by different letters are significantly different (P < 0.05).

** P < 0.01

*** P < 0.001.

A: Site A; D: Site D; H: Site H; I: Site I; C: Bulk soil; M: Rhizosphere soil.

**Table 4:** Estimated number of observed operational taxonomic units (OTUs), coverage, richness, and diversity at three types of dunes.

Samples	OTU	Coverage (%)	ACE	Chao1	Shannon
EC	867.67a	99.21ab	956.47b	935.43b	2.80c
EM	464.67b	99.62a	533.79c	516.87c	3.48ab
FC	902.00a	99.25ab	948.47b	910.72b	2.85c
FM	1212.67a	98.91b	1166.36a	1155.68a	4.02a
GC	940.67a	98.88b	1053.11ab	1043.37ab	3.08bc
GM	1240.00a	99.23ab	1189.67a	1174.01a	3.50ab
*F*	10.05	4.21	34.42	32.31	5.99
*P*	**	*	***	***	*

OTUs, operational taxonomic units; Coverage, Good's nonparametric coverage estimator; ACE, abundance-based coverage estimator; Chao1, richness estimator; Shannon, nonparametric Shannon diversity index.Values are means of three replicates.

Means in the column followed by different letters are significantly different (P < 0.05).

* P < 0.05

** P < 0.01

*** P < 0.001.

E: Fixed dune; F: Semi-fixed dune; G: Moving dune; C: Bulk soil; M: Rhizosphere soil.

**Fig 4:**
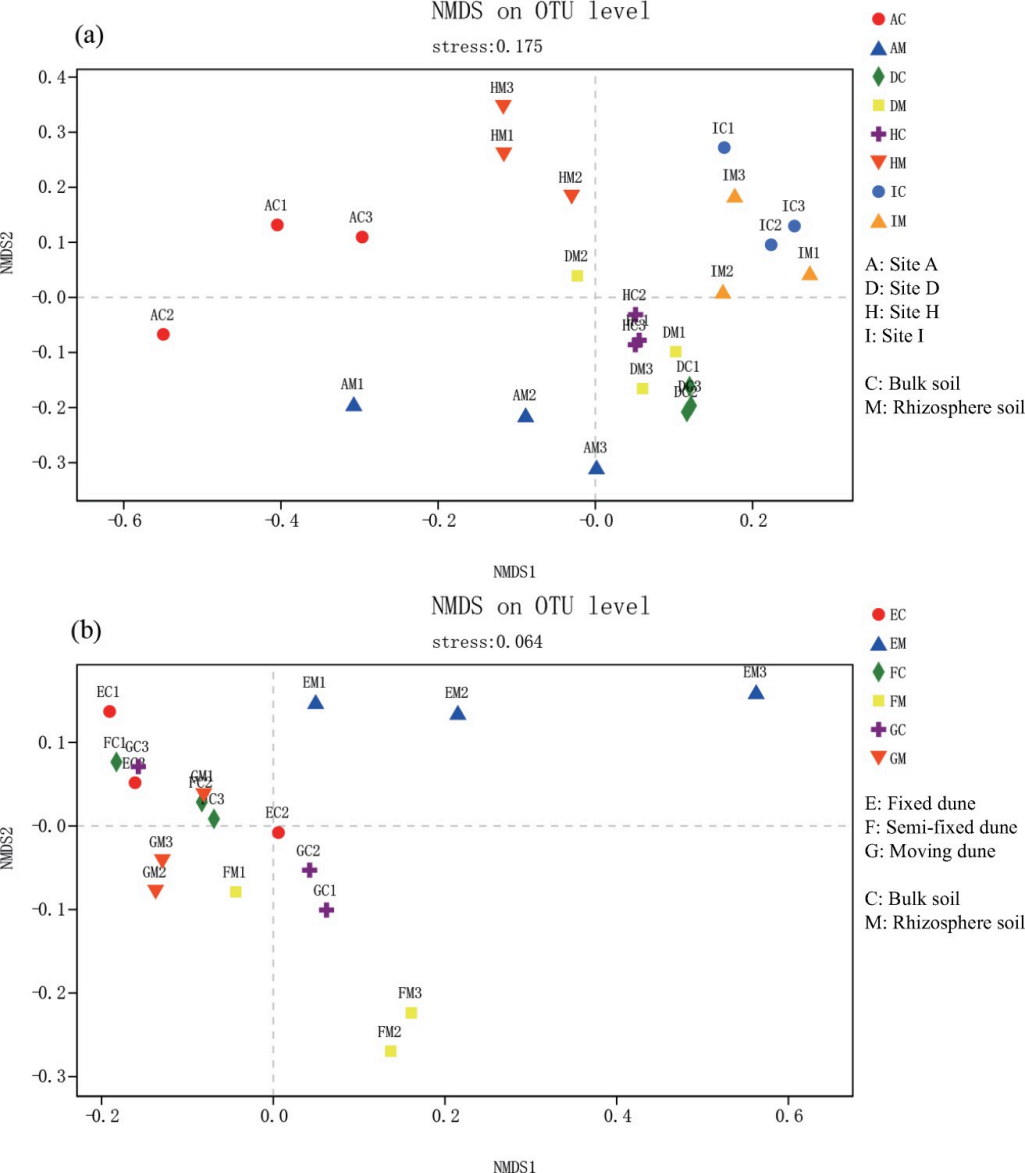
Analysis of soil bacterial community composition by non-metric multi-dimensional scaling (NMDS) based on Bray-Curtis distances at operational taxonomic units (OTU) levels. Across (a) four sites and (b) three types of dunes.

NMDS analysis based on Bray-Curtis distances at the OTU level revealed similarities between soil bacterial communities at different sites and different types of dunes (Fig 4). The results of PERMANOVA (based on Bray-Curtis distances at OTU levels) demonstrated that the response of soil bacterial community to AI (F = 3.906, P = 0.001) was more sensitive than that of dune type (F = 0.850, P = 0.517). The bacterial community structure showed significant differences between rhizosphere and bulk soil on dunes of different types (F = 5.189, P = 0.004), and no significant difference at sites with different AI (F = 0.062, P = 0.955).

### Correlation between bacterial communities and soil properties

Before analyzing the correlation between the soil bacterial community data and environmental factors, variance inflation factor (VIF) analysis was used to screen environmental factors and the threshold was usually set to 10. Environmental factors including EC (VIF = 4.41), TOC (VIF = 3.75), TN (VIF = 7.76), TP (VIF = 5.74), K^+^ (VIF = 6.89), and Na^+^ (VIF = 2.22) were selected at different sites. Environmental factors pH (VIF = 1.73), EC (VIF = 3.75), TOC (VIF = 1.39), TP (VIF = 3.42), and K^+^ (VIF = 2.17) were selected at different types of dunes.

**Fig 5:**
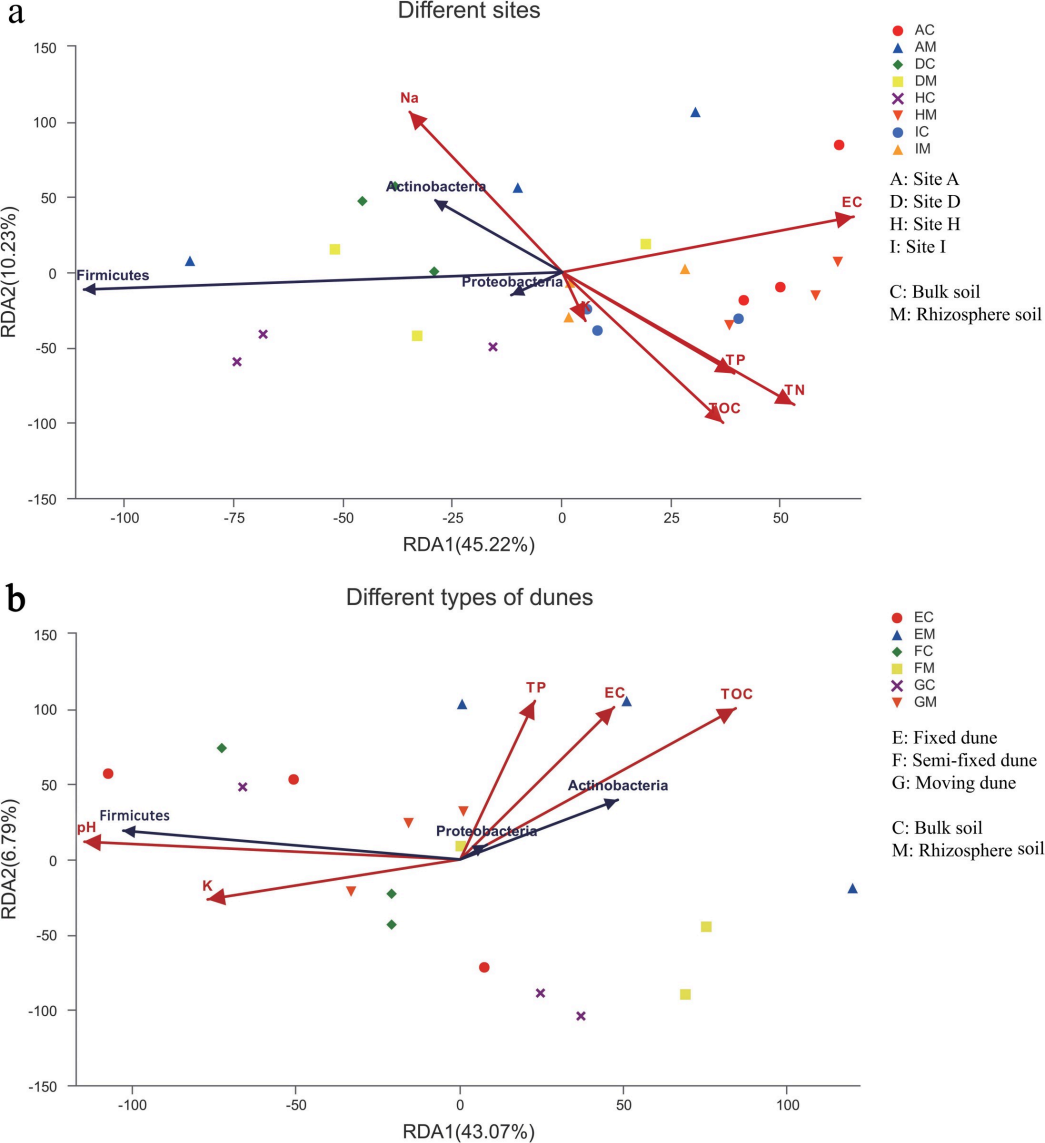
Redundancy analysis (RDA) of abundant phyla and soil properties for individual soil samples. From (a) four sites and (b) three types of dunes.

Results of the RDA showed a significant correlation between the soil properties and dominant phyla. The first two RDA axes explained 55.45% and 49.86% of the total variation at four sites (Fig 5A) and three types of dunes (Fig 5B). The results revealed that soil TOC, TN, and Na^+^ significantly affected bacterial communities at different sites while TP also played a significant role (Table 5). Soil pH and TP had significant effects on bacterial communities at different types of dunes, but EC and TOC had more significant effects (Table 6).

**Table 5:** Correlation between soil properties parameters and redundancy analysis (RDA) axes at four sites.

Soil properties	RDA1	RDA2	R^2^	P value
EC	0.903	0.430	0.176	0.145
TOC	0.424	−0.906	0.463	0.004**
TN	0.546	−0.838	0.441	0.004**
TP	0.537	−0.843	0.253	0.041*
K^+^	0.286	−0.958	0.042	0.648
Na^+^	−0.399	0.917	0.509	0.002**

* P < 0.05.

** P < 0.01.

**Table 6:** Correlation between soil properties parameters and redundancy analysis (RDA) axes at three types of dunes.

Soil properties	RDA1	RDA2	R^2^	P value
pH	−0.952	−0.305	0.406	0.018*
EC	0.677	0.736	0.447	0.009**
TOC	0.754	0.657	0.700	0.001**
TP	0.588	0.809	0.352	0.035*
K^+^	−0.870	−0.493	0.267	0.096

* P < 0.05.

** P < 0.01.

**Fig 6:**
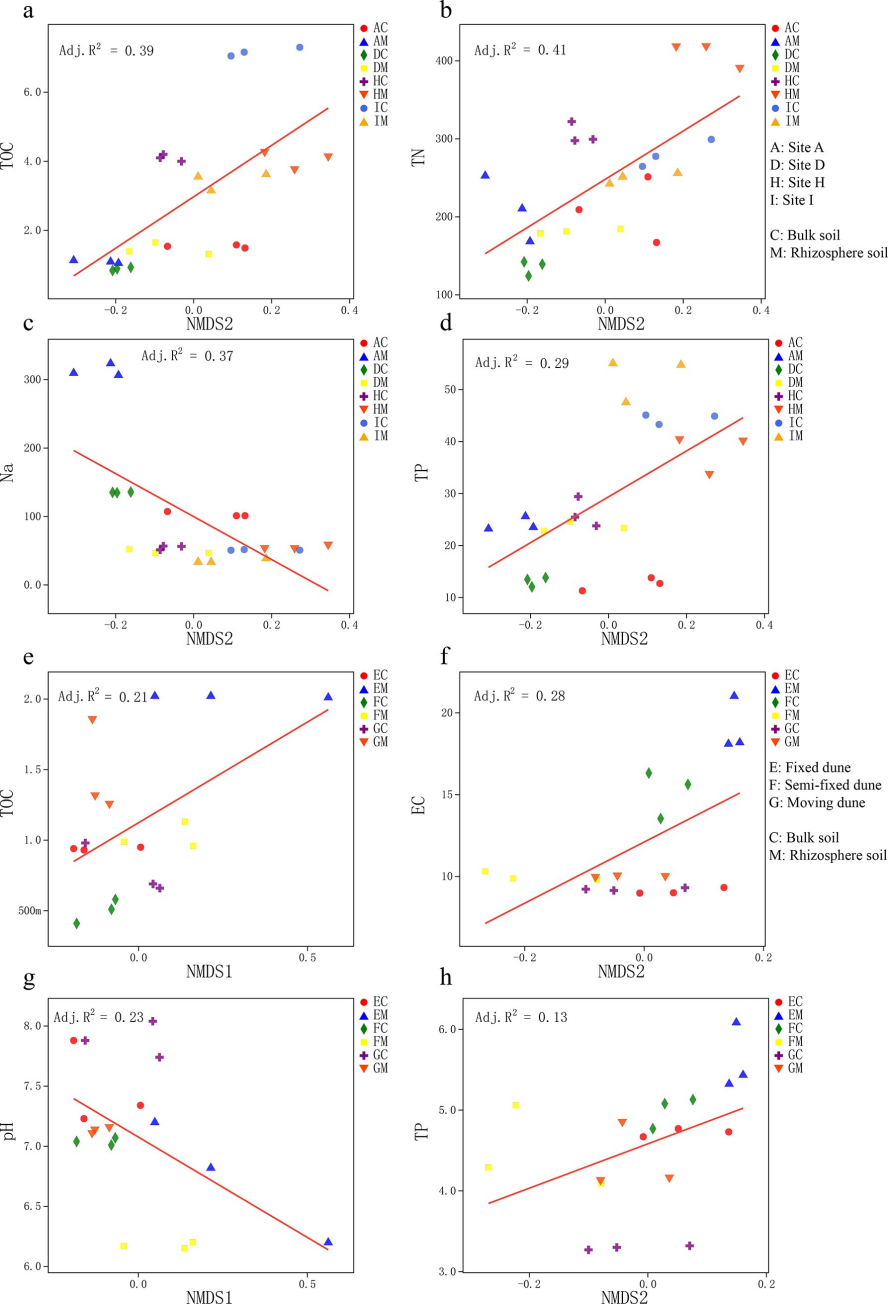
Linear regression analysis of soil samples. The strongest correlation of each non-metric multi-dimensional scaling (NMDS) axis is given: (a) NMDS2 vs total C (TOC), (b) NMDS2 vs total N (N), (c) NMDS2 vs Na^+^, and (d) NMDS2 vs total P (TP). Lines of best fit from linear least-squares regression at different sites: (e) NMDS1 vs TOC, (f) NMDS2 vs EC, (g) NMDS1 vs pH, and (h) NMDS2 vs TP. Lines of best fit from liner least-squares regression at different types of dunes.

**Fig 7:**
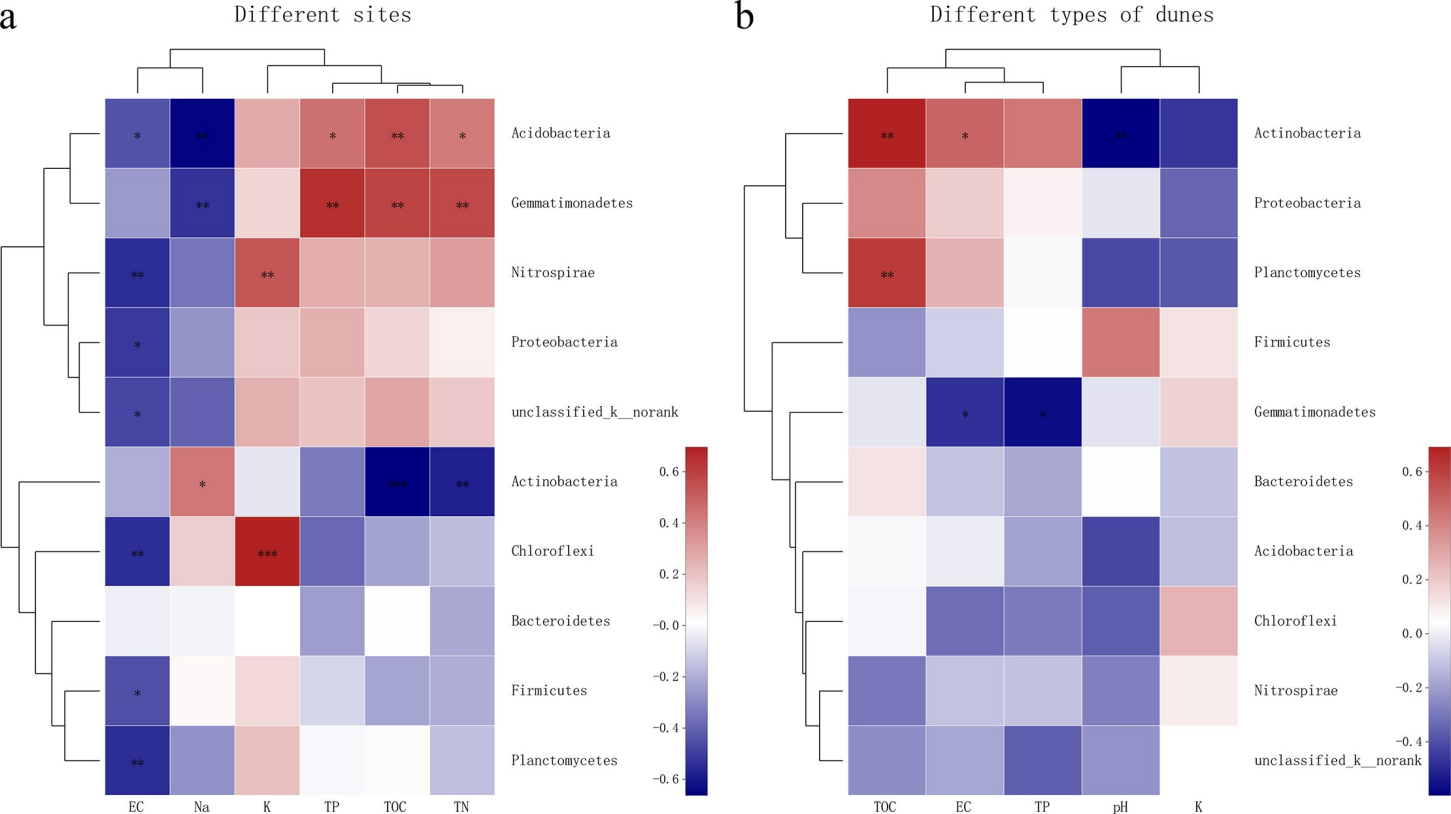
Spearman correlation heatmap of bacterial communities and soil properties. Across (a) four sites and (b) three types of dunes. R values are shown in different colors and illustration on the right is color range of different R values. *P < 0.05, are marked with **P < 0.01, and ***P < 0.001.

The linear regression analysis showed that the changes between bacterial communities and TOC, TN, TP at different sites were consistent on the axis of the NMDS2, but opposite to Na^+^ (Fig 6A–6D). At different types of dunes, the changes in bacterial community were consistent with TOC (vs NMDS1), EC (vs NMDS2), and TP (vs NMDS2), and opposing to pH (vs NMDS1, Fig 6E–6H). According to the Spearman correlation analysis, the dominant phyla Acidobacteria, Gemmatimonadetes, and Actinobacteria were significantly affected by environmental factors at different sites (Fig 7A). Acidobacteria and Gemmatimonadetes were significantly positively correlated with TOC, TN, and TP, while significantly negatively correlated with Na^+^. However, Actinobacteria was significantly negatively correlated with TOC and TN, and positively correlated with Na^+^. Firmicutes and Proteobacteria, which were the main components in all soils, were only negatively correlated with EC. Actinobacteria, Planctomycetes, and Gemmatimonadetes, the dominant phyla at different types of dunes were strongly influenced by environmental factors (Fig 7B). Actinobacteria was significantly positively correlated with TOC and EC, and significantly negatively correlated with pH. TOC was also significantly positively correlated with Planctomycetes while Gemmatimonadetes was negatively correlated with EC and TP.

## Discussion

### Soil properties were affected by rhizosphere, aridity, and dune type

According to Tables 1 and 2, soil pH of rhizosphere was generally lower than that of the bulk soil, especially in different types of dunes. This could be attributed to the release of organic acids into the soil by the secretion of plant roots, decomposition of organic matter, and metabolism of microorganisms [38,39]. The main factor affecting pH was found to be rhizospheres in different types of dunes, however, aridity determines the variation trend of pH at different sites. Variation of soil nutrient contents such as TOC, TN, and TP reflected the transition from arid to semi-humid regions, and from moving to fixed dunes. However, rhizosphere was still the main factor affecting soil nutrient content, which was more significant in arid regions and fixed dunes. Soil ion contents such as Ca^2+^, K^+^, and Na^+^ in rhizosphere soil showed a downward trend with the increase of AI. Moreover, the content of ions in rhizosphere soil of different types of dunes was significantly lower than bulk soil. Studies have shown that the active absorption of Ca^2+^, K^+^, and Na^+^ in soil by plant roots could promote the synthesis of proteins related to drought resistance, thus, promoting the growth of plants in arid areas [40–42]. We believe that in order to adapt to different desertification environments, *C*. *microphylla* strengthened the root's absorption of soil ions, resulting in the decrease of rhizosphere ion content.

### Bacteria responded to rhizosphere, aridity and dune type

The bacterial community composition and diversity reflected the bacterial response to rhizosphere, aridity, and dune type (Fig 2A and 2D). Firmicutes, Actinobacteria, Proteobacteria, Acidobacteria, Chloroflexi, Bacteroidetes, and Planctomycetes were the most dominant phyla at all study area. Other studies of soil bacteria have confirmed that these phyla were also a major component [43]. The relative abundance of some dominant phylum changed significantly under the influence of rhizosphere, aridity, and dune type (Fig 2B, 2C, 2E and 2F). Firmicutes and Actinobacteria were the most dominant phyla among all the samples in this study, and they are the main components in arid soil worldwide [44]. Some researchers have reported that they were well adapted to environmental stresses such as drought and high salt content, and could survive in barren soil [45,46]. The relative abundance of Firmicutes and Actinobacteria varies significantly between rhizosphere and bulk soil of different types of dune, which reflects their adaptation to the environment (Fig 2E). The relative abundance of Bacteroidetes in the rhizosphere at different sites was significantly higher than that in the bulk soil (Fig 2C), this result represented the improvement of rhizosphere soil conditions [47]. At four sites with different AI, Proteobacteria were found at a higher proportion in rhizosphere soil of relatively humid areas (Fig 2B). This was because Proteobacteria were a group of gram-negative bacteria with a rapid reproduction rate, and the nutrient-rich environment was more suitable for the proliferation of this phylum, and many members of this phylum were responsible for fixing nitrogen and producing plant-friendly polycyclic aromatic hydrocarbons [48]. The variation trend of relative abundance of Acidobacteria among the four sites was similar to that of Proteobacteria, but we believe that the reasons for this result were different (Fig 2B). From site A to site I, the pH value decreases successively, while acidic soil was more suitable for the proliferation of Acidobacteria. Recent studies had also confirmed that Acidobacteria contains plant-promoting bacteria that promote plant growth [49]. Other studies had found that the increase of Acidobacteria were mainly due to the presence of Actinomycetales members [50].

At the genus level, *Lactococcus*, *Bacillus*, *Pseudarthrobacter*, *Solibacillus*, and *Pseudomonas* were the most dominant genera in all soils (Fig 3A and 3D). *Bacillus* had been proven to have a variety of plant growth promoting functions in numerous studies [51], accounting for the abundance in all rhizosphere soils. *Pseudomonas* also constituted a high proportion of the rhizosphere soil bacterial community. This species colonizes the soil adequately and survives under different stress conditions because it grows rapidly, uses various substrates as nutrients, and produces various compounds that promote the growth of plants [10]. Some studies in arid and semi-arid regions suggest that the composition of plant associated microbial communities was mainly influenced by plant compartment [28]. However, our research results showed that the relative abundance of the most dominant genera, such as *Pseudarthrobacter*, *Streptomyces* and *norank_o__Gaiellales*, was more significantly affected by geographical location (Fig 3B and 3C). The major genera were significantly affected by rhizosphere in different types of dune, but the dune types had no significant influence on them (Fig 3E and 3F). The significant difference in relative abundance of these dominant genera between rhizosphere and bulk soil, and the more abundant unique OTUs in rhizosphere soil (S2 Fig), indicated that the plant root system had established certain selection pressure on the rhizosphere soil bacterial community [52].It is worth noting that many genera suck as *Streptomyces*, *Jatrophihabitans* and *norank_o__Gaiellales* had higher relative abundance in rhizosphere than in the bulk soil. These genera were not the most dominant genera, but these bacterial groups may play an important role in the growth of plants in desert environments [53].

Soil bacterial diversity was considered to be critical to the integrity, function, and long-term sustainability of soil ecosystems. Therefore, the higher diversity of soil bacteria could make soil ecosystems more stable [54,55]. In this study, with the increase in AI, soil properties were more conducive to the proliferation of bacteria, the total amount of OTUs in soil increased, and AI also significantly affected the diversity and richness of rhizosphere soil bacteria at different sites (Table 3). Yet research suggests that drought does not seem to have much effect on the phylogenetic diversity of soil bacterial communities [56]. At different types of dunes, the diversity and richness of bacteria in rhizosphere soil were significantly higher than those in bulk soil were (Table 4). The bacterial community in rhizosphere was mainly derived from bulk soil, and the host specific bacterial communities were enriched, so the diversity in rhizosphere soil was usually decreased [57]. However, the condition of bulk soil in this study was inferior to that of rhizosphere soil, the rhizosphere provided more favorable conditions for bacterial enrichment and reproduction, which may be the main reason for the higher diversity in rhizosphere soil than in bulk soil.

The substances secreted by roots might have provided abundant nutrients for the bacteria, thus recruiting more bacteria. The bacterial community structure of the soil was mainly affected by the aridity of the different sites. However, it was affected by rhizosphere to varying degrees at each site, and the effect was more significant at the drier regions than it was at other regions (Fig 4A). Similarly, rhizosphere was also a major factor affecting bacterial communities in different types of sandy dunes (Fig 4B). Combined with bacterial community composition and PERMANOVA analysis results, we believe that at four sites, the effect of AI on bacterial community was stronger than rhizosphere. In three different types of dune, rhizosphere had stronger influence on bacterial community than dune type. Drought may affect rhizosphere bacterial communities by modulating moisture availability, changing soil physicaochemical properties and plant phenotypes [14]. The colonization of *C*. *microphylla* in different types of dune changes the stability and physicaochemical properties of soil, which has a strong influence on rhizosphere bacterial community

### Investigation of factors influencing bacterial community structure and distribution

The results suggested that under the influence of different aridity and dune type, the response of bacterial community to environmental factors changed (Figs 5 and 6, Tables 5 and 6). The main phyla Firmicutes, Actinobacteria, and Proteobacteria were significantly negatively correlated with soil nutrient content at different sites, whereas at different types of dunes they were significantly positively correlated with soil nutrient content. Soil Na^+^ and K^+^ contributed greatly to changes in the bacterial communities, in particular, they were positively correlated with Firmicutes, which was the phylum with the highest abundance, consistent with findings of other studies [58]. The correlation analysis of soil properties and the RDA axis in our study revealed that TOC, TN, and Na^+^ had the most significant influence on the bacterial community at different sites. At different types of dunes, TOC and EC were the mainly factors influencing the bacterial community, followed by pH and TP. Rhizosphere bacterial communities in desertification environments were determined by how different desertification environments shape host plants and surrounding soil. Changes in the soil nutrient cycle in different desertification environments and the resulting changes in soil bacterial communities will have an impact on plant health because plants rely on bacterial activity to make soil nutrients biologically available. At the same time, the changes of root exudates caused by desertification conditions may change the composition and activity of surrounding soil bacterial communities and promote the further change of soil physicaochemical properties [14].

Spearman correlation analysis showed that the dominant bacteria most strongly affected by environmental factors at different sites were Acidobacteria, Gemmatimonadetes, and Actinobacteria, among which Acidobacteria and Gemmatimonadetes were significantly negatively correlated with Na^+^ content (Fig 7A). This suggests that these phyla may be related to the uptake of Na^+^ by the root system in rhizosphere soil, which contributed to improving the resistance of plants to drought and high salt stress [40,59]. Soil EC was a major factor affecting the structure and distribution of soil bacterial community in many studies [60]. It has an impact on many dominant phyla, especially Firmicutes and Proteobacteria, which was consistent with the findings of other studies [61,62]. At different types of dunes, Actinobacteria, Planctomycetes, and Gemmatimonadetes were particularly affected by environmental factors (Fig 7B). The effect of soil pH on soil bacterial community was mainly identified by the effect on Actinobacteria. The increased abundance of Actinobacteria in the rhizosphere soil was mainly caused by the decrease in soil pH caused by organic acids secreted by the root system [63]. These rhizosphere bacterial communities with strong response to the environment may be closely related to plants' adaptation to adversity. To accurately understand how drought and dune types affect rhizosphere bacterial communities is an important step to formulate strategies to combat desertification [14].

## Conclusion

In this study, the soil properties and bacterial communities of rhizosphere and bulk soils of *C*. *microphylla* from desertification regions of northern China were investigated and compared. The results indicated that compared with the bulk soil, the rhizosphere soil nutrient content was generally improved in different desertification environments, for example, the content of TP was significantly higher than bulk soil. Furthermore, the richness and diversity of bacterial communities were significantly higher than that in bulk soil. Our study illustrates that the structure of the soil bacterial community was affected by rhizosphere, aridity, and dune type. The relationship between the bacterial community and environmental factors was revealed. These results provide a solid piece of information necessary for a better understanding of the role of different aridity index or dune types in altering soil bacterial community of rhizosphere of *C*. *microphylla*, which is extremely crucial for the vegetation restoration in desertification areas.

## Supporting information

S1 Fig Rarefaction curves based on observed species value. (TIF)

S2 Fig Venn diagram showing unique and shared OTUs between (a) all rhizospheric soil and bulk soil at four sites, (b) all rhizospheric soil and bulk soil at three types of dunes, (c) each rhizospheric soil at four sites,(d) each rhizospheric soil at three types of dunes.
(TIF)
