# Supplementary figures and images for: Effect of aridity and dune type on rhizosphere soil bacterial communities of Caragana microphylla in desert regions of northern China
Source: PLoS One. 2019 Oct 18;14(10):e0224195. doi: 10.1371/journal.pone.0224195 (PMC6799922; doi:10.1371/journal.pone.0224195)

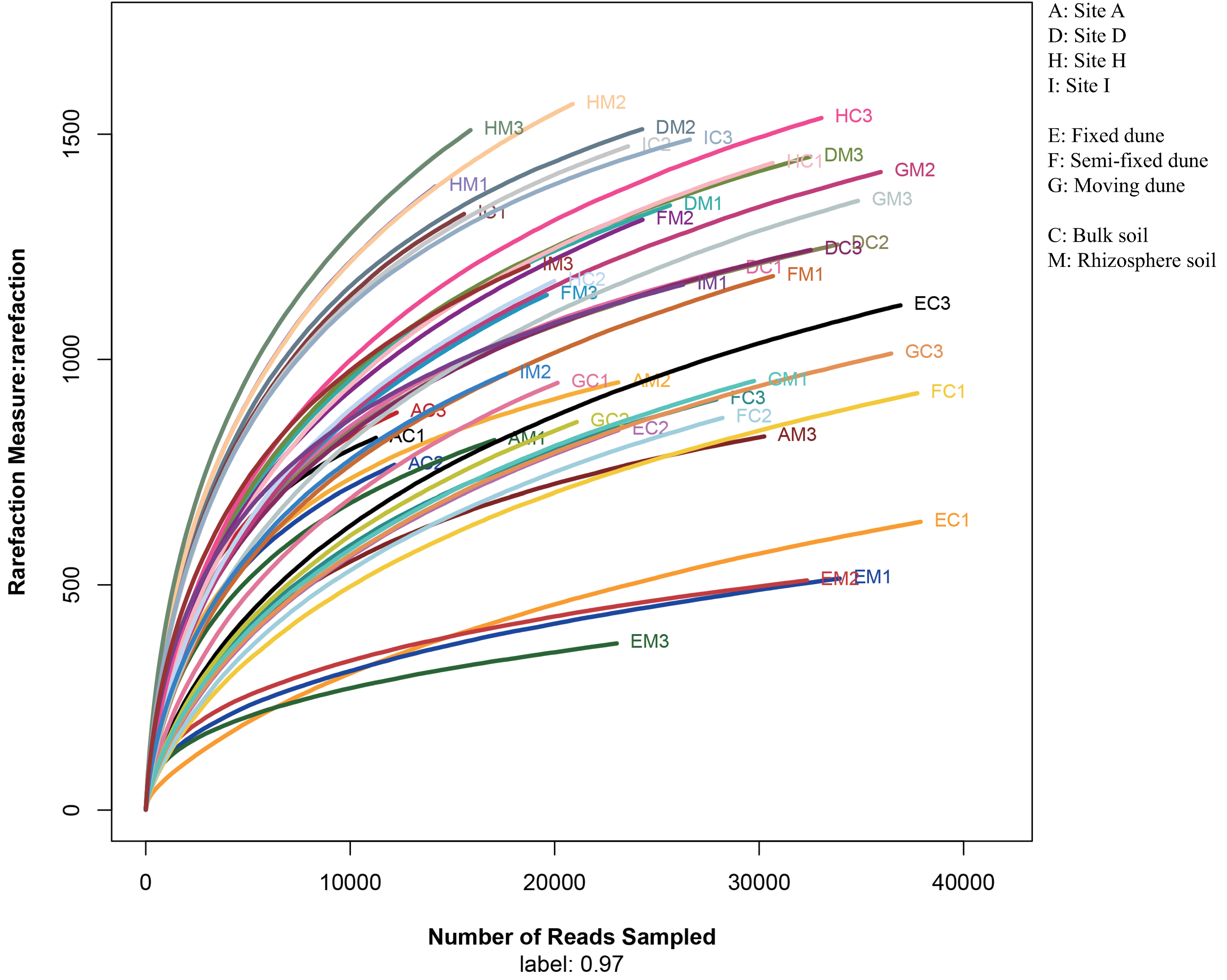

Supplement: S1 Fig — (TIF) [file pone.0224195.s001.tif]

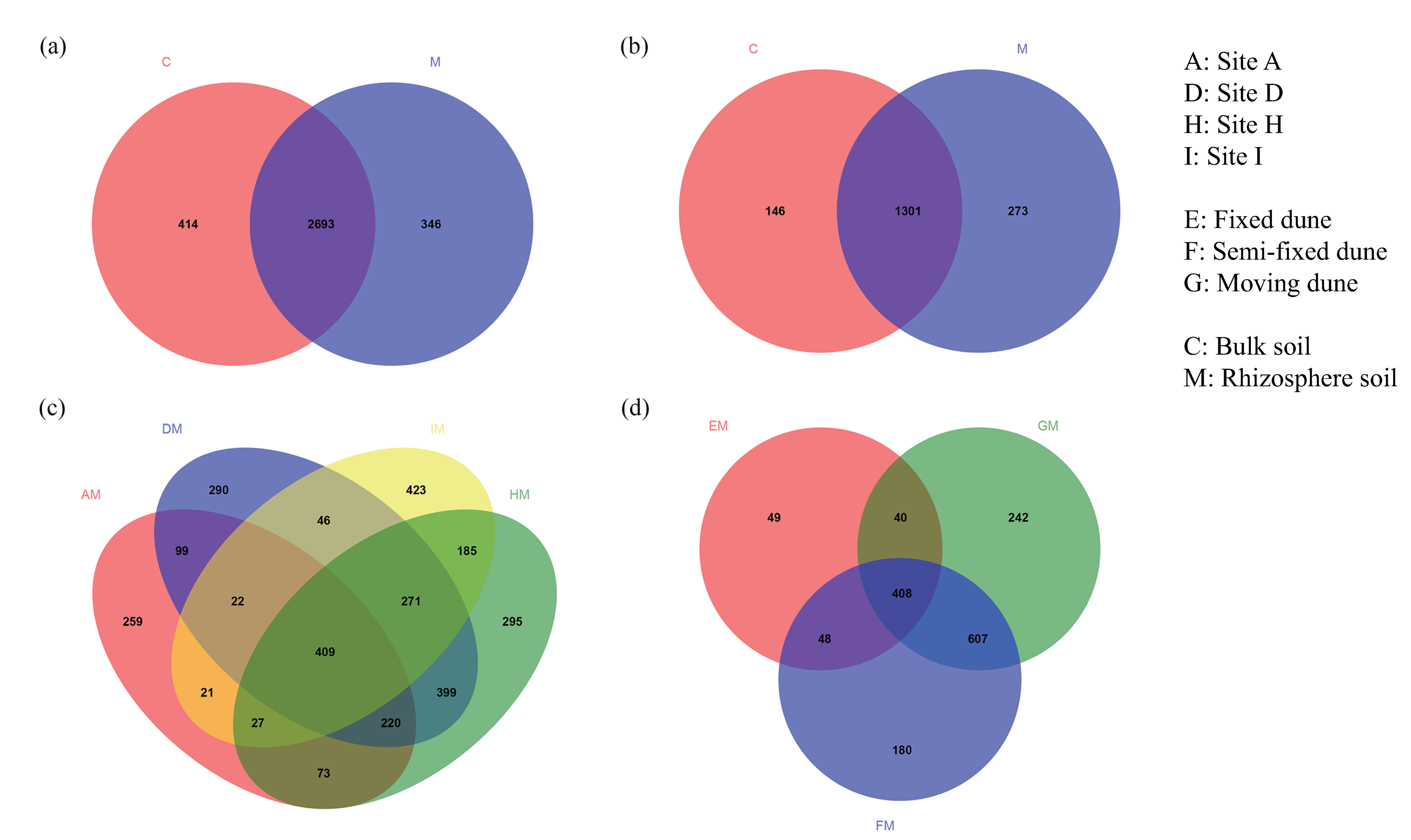

Supplement: S2 Fig — Venn diagram showing unique and shared OTUs between (a) all rhizospheric soil and bulk soil at four sites, (b) all rhizospheric soil and bulk soil at three types of dunes, (c) each rhizospheric soil at four sites,(d) each rhizospheric soil at three types of dunes. (TIF) [file pone.0224195.s002.tif]
